# Prevalence of diagnosed diabetes in an urban area of Puducherry, India: Time for preventive action

**DOI:** 10.4103/0973-3930.50708

**Published:** 2009

**Authors:** Anil J. Purty, D. R. Vedapriya, Joy Bazroy, Sanjay Gupta, Johnson Cherian, Mohan Vishwanathan

**Affiliations:** Department of Community Medicine, Pondicherry Institute of Medical Sciences (A Unit of Madras Medical Mission), Kalapet, Pondicherry-605 014, India; 1 Madras Diabetes Research Foundation, Chennai, India

**Keywords:** Community-based study, diabetes prevalence, undiagnosed diabetes, urban Puducherry

## Abstract

**BACKGROUND:**

Epidemiological studies in India have shown that the prevalence of diabetes in the population is at least twice the number of persons diagnosed with diabetes residing in the given area. Similarly, community-based prevalence figures are unavailable in Puducherry,.

**AIMS:**

The aim of this study was to estimate the number of persons diagnosed with diabetes mellitus in Puducherry.

**SETTING AND DESIGN:**

This study was conducted in the service area of the Urban Health Centre (UHC), Pondicherry Institute of Medical Sciences (PIMS), Puducherry with retrospective data from family records.

**METHODS:**

The diagnosis of diabetes was retrospectively documented by reviewing all family folders of 2667 families (Population 11,835) for the period of 1/1/2003 to 31/12/2006. The data was verified by home visits from January until March, 2007. The *case definition used, was a resident diagnosed with diabetes by a medical doctor and who was on antidiabetes treatment for at least the past six months.*

**RESULTS:**

We found 643 individuals who had been diagnosed with diabetes. The prevalence of known diabetes was estimated to be 5.6% (5.31% in males and 6.1% in females). The age-sex specific prevalence was estimated using the 2001 Census data. There are about 48,876 known diabetics living in Puducherry.

**CONCLUSIONS:**

(1) Community-based health surveillance data comprise a useful tool to measure the prevalence of diagnosed cases of diabetes mellitus within the Indian context; 2) Diabetes mellitus is an important public health priority requiring urgent preventive action as there are about 97,752 persons in Puducherry who have either been diagnosed with diabetes or remain undiagnosed for the disease.

## Introduction

The prevalence of diabetes is rapidly rising all over the world.[1] Current estimates are that there are at least 150 million people living with diabetes worldwide of which two-thirds are in developing countries.[2] The total number of people with diabetes is predicted to rise above 300 million by 2025. The largest increase of the diabetic population occurs in the most economically productive age group.[3] Over the past three decades, diabetes has become a major cause for morbidity and mortality affecting the youth and the middle-aged. Although the prevalence of type 1 diabetes is also increasing, type 2 diabetes accounts for more than 90% of all the diabetes cases.

India is being called the diabetic capital of the world, with over 30 million diabetic individuals. Population-based studies showing the prevalence of type 2 diabetes in different parts of India have recently been reviewed[4,5] and shows that the prevalence has risen five-fold from 2.1% in 1975 to 12.1% in 2000.[6–13] The CUPS (Chennai Urban Population Study) and CURES (Chennai Urban Rural Epidemiological Study) showed age-standardized prevalence rates of 12% for urban India. However, there have been no community-based prevalence rates for diabetes available from Puducherry in the recent past except for a hospital-based study[14] and a few lay reports[15] that indicated that there is an increasing problem of diabetes in the region. It is important to have region-specific prevalence data of diabetes so that appropriate public health measures can be initiated by public policymakers and supported by all those concerned.

It has been noted that the studies which have shown an increase in prevalence of diabetes have also reported a very high prevalence of undiagnosed diabetes in the community. The CURES study showed an age-standardized prevalence rate of 14.3% whereas the CUPS study showed 9.3%. The overall prevalence of diabetes in the CUPS study was reported to be 12%.[16–18] Similarly, in the Amrita Diabetes and Endocrine Population Survey (ADEPS) study from Kerala, the prevalence of known and undiagnosed diabetes was 9.0 and 10.5% respectively.[19] A study from Kashmir showed that the prevalence of undiagnosed diabetes was 4.25%, which was more than double of that of known diabetes (1.9%).[20]

Thus, in estimating the prevalence of diabetes in a defined geographical area, the logical preliminary step would be to know the number of diagnosed diabetes patients currently on treatment and then apply community-based, cost-effective screening methods among the high-risk group to identify the undiagnosed population. Several community-based interventional studies have shown that early diagnosis and treatment not only delay the onset of complications,[21,22] but also, more importantly, indicate that the onset of diabetes can be prevented or delayed by lifestyle interventions among those identified to be at risk for developing diabetes.[23–25]

As a first step towards achieving this end, we undertook this study to estimate the prevalence of known diabetes in the service area of the Urban Health Centre (UHC), Pondicherry Institute of Medical Sciences (PIMS) in Muthialpet, and then use this data to estimate the number of persons diagnosed with diabetes in the State of Puducherry.

## Materials and Methods

The Union Territory of Puducherry has an area of 480 sq. km. and a population density of 2,034 per sq. km. (as against the national average of 324 per sq km).[26] The 2001 census showed the population of Puducherry to be 9,72,432, (4,87,053 males and 4,86,379 females).[27]

The service area of the UHC PIMS in Muthialapet (urban Puducherry) covers the population living in the 12 sq km area between Salai Street and Thidal Street along the East Coast Highway that links Puducherry to Chennai. This typical urban area in the region has 2677 resident families with a population of 11,835 (5934 males, 5901 females).

**Figure 1:**
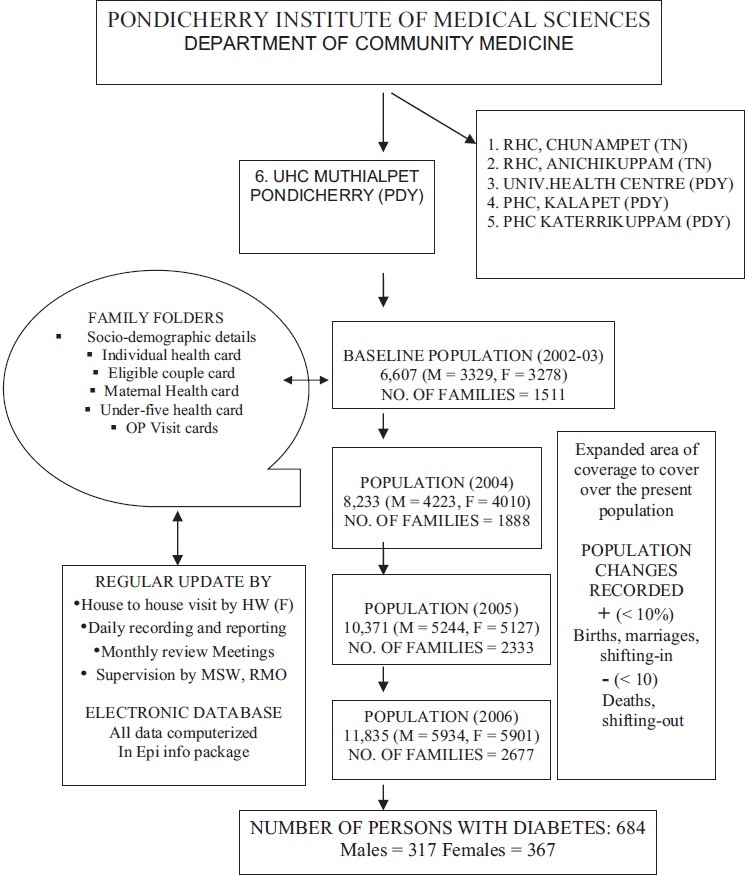
Flow chart showing method of data collection

Figure 1 shows the health surveillance system in the PIMS UHC service area. The family folder system at PIMS was started in 2002 to record the socioeconomic and health data of each family living in the service area. The Muthialpet service area has been subdivided into three areas, A, B, and C consisting of 900–1000 families each. One female health worker is assigned to cover each area once in three months by visiting at least 15 houses per day. A medical social worker (MSW) visits the area for supervision every week. The medical officer and PIMS faculty members make a supervisory visit and a priority visit once or twice every month. The family folder is a record filing system containing the sociodemographic details of each family member and information on individual health, maternal health care, under-five health status, and records of all eligible couples. Every week, on Saturdays, the changes recorded during the home visits (migrations, births, deaths, marriages etc) are electronically updated using Epi-Info software and reviewed at the monthly meeting in the department.

The family folders of all 2677 families between 1/1/2003 and 31/12/2006 were reviewed to collect information on the persons with known diabetes [Figure 1]. The name, age, sex, and residential address of individuals were noted along with documented evidence of treatment of diabetes. This list was verified by home visits during the period of January to March 2007 and the concerned individuals or their family members were asked regarding the treatment for diabetes. All those individuals who confirmed that they were diagnosed with diabetes by a medical doctor and who had some documented evidence of treatment for diabetes were included in this study. Those who could not be contacted for verification either because of nonavailability, shifting of residence, death, had stopped treatment for diabetes, or were on treatment for less than six months were not included in this study.

The study protocol was approved by the PIMS Institutional Ethics Committee. Informed consent was obtained from the individuals before their inclusion, thus ensuring confidentiality. They were encouraged to continue regular treatment and follow-up for their illness. We listed 768 individuals with diabetes from our records and were able to verify the diagnosis of diabetes in 684 (89%) persons.

## Results

Table 1 shows the age-specific prevalence of known diabetes in the area. There were a total of 684 individuals with diabetes in the population of 11,835, the prevalence was found to be 5.8%. In the age group of ≥ 20 years, the prevalence was 8.2% whereas the age-specific prevalence was above 20% after the age of 50 years. The youngest diabetic child was an 11 month-old male baby with type 1 diabetes.

**Table 1:** Age-specific prevalence of known diabetes in PIMS UHC Muthialpet, 2006

Age group (Yrs)	Population (%)	No. of persons with DM	Age-specific prevalence(%)	
0–9	1695 (14.3)	2	0.12	Prevalence of DM in Population (aged ≥ 20 years)
10–19	1909 (16.3)	6	0.31	
20–29	2210 (18.6)	18	0.81	
30–39	2172 (18.4)	35	1.61	
40–49	1612 (13.6)	84	5.21	
50–59	1136 (9.6)	226	19.89	
60–69	710 (5.9)	214	30.14	
70–79	288 (1.9)	76	33.33	
≥ 80	112 (0.9)	23	20.54	
Total	11,835 (100)	684	*5.8%	**8.2%

* General Prevalence,

** Prevalence in population over 20 years of age

Table 2 shows the age-sex specific prevalence of known diabetes in the area. There were 317 (46%) males and 367 (54%) females with known diabetes. The prevalence rates were 5.3% among males and 6.2% among females; the difference was not statistically significant (*P* > 0.05). In the age group of 20–29 and 50–59 years, there were more females with diabetes and this difference was statistically significant. (*P* < 0.05)

**Table 2:** Age-sex specific prevalence of known diabetes in PIMS UHC, Muthialpet, 2006

Age group	Males			Females		
		
(Yrs)	Population (%)	No. with DM	Age-specific prevalence (Males) (%)	Population (%)	No. with DM	Age-specific prevalence (Females) (%)
0–9	861 (14.5)	1	0.12	834 (14.2)	1	0.12
10–19	962 (16.2)	3	0.31	947 (16.1)	3	0.32
20–29	1035 (17.4)	2	0.19	1166 (19.8)	16	1.37
30–39	1098 (18.5)	14	1.27	1074 (18.2)	21	1.95
40–49	860 (14.5)	42	4.88	752 (12.7)	42	5.58
50–59	552 (9.3)	93	16.85	584 (9.8)	133	22.77
60–69	341 (5.7)	115	33.72	369 (6.4)	99	26.83
70–79	170 (2.9)	34	20	118 (1.9)	42	35.59
≥ 80	55 (0.9)	13	23.63	57 (0.9)	10	17.54
Total	5,934 (100)	317	5.34	5,901 (100)	367	6.15

Table 3 shows the estimated number of diagnosed diabetics in Puducherry based on the 2001 census data for the State. In a population of 9,72,432, there are about 48,876 persons with diabetes using the prevalence estimate of 5.8%. The largest numbers of diabetics (58.4%) appear to be in the age groups of 50–59 (13,670) and 60–69 (14,800) years.

**Table 3:** Estimated number of known diabetics in Puducherry based on Census data, 2001, by age-specific prevalence rates

Age group (Yrs)	Males	Females	Total Population(%)	Age-specific prevalence of DM in study area (%)	Estimated No. persons with DM in UT of Pondicherry (%)
0–9	82,600	85,670	1,67,270 (17.2)	0.12	200 (0.4)
10–19	94,615	97,389	1,92,004 (19.7)	0.31	595 (1.2)
20–29	1,00,207	94,002	1,94,209 (19.8)	0.81	1573 (3.2)
30–39	78,741	80,899	1,59,640 (16.4)	1.61	2570 (5.3)
40–49	51,826	57,738	1,09,564 (11.3)	5.21	5708 (11.7)
50–59	34,509	34,220	68,729 (7.2)	19.89	13670 (28.1)
60–69	26,894	22,209	49,103 (5.2)	30.14	14800 (30.3)
70–79	13,097	10,600	23,697 (2.4)	33.33	7892 (16.1)
≥ 80	4,564	3,652	8,216 (0.8)	20.54	1868 (3.7)
Total	4,87,053	4,86,379	9,72,432 (100)	*5.8%	48,876 (100)

## Discussion

The first nationwide study in India was performed by the Indian Council of Medical Research Task Force[28] on diabetes in which, 34,194 subjects were screened and the prevalence of diabetes was shown to be 2.1% in urban subjects and 1.5% in the rural population. There has been a rapid increase in diabetes epidemiology studies in India in the past 20 years. Initial studies among urban Delhi residents reported the prevalence of known diabetes and compared this with the diabetes prevalence in Southall, London.[29]

In large Indian cities like Chennai,[7,10,11] Trivandrum,[12,13] Mumbai,[30] Delhi,[31] Jaipur,[32] and Guwahati,[19] as well as in a national study in large metropolises[33] and industrial populations,[34] diabetes prevalence among adults (≥ 20 years) has ranged from 8 to 15%. Our study shows the prevalence of known diabetes to be about 5.8% in the general population and 8.2% in the age group above 20 years. Screening for diabetes is likely to double the number of patients.

There is a large heterogeneity of diabetes prevalence within urban populations depending on the socioeconomic stratum studied and the sampling response rates. In the late 1990s, Ramachandran *et al.* and Mohan *et al*. reported a high prevalence of diabetes (11–12%) in Chennai.[7,18,36,37] Our study is similar to the prevalence of known diabetes shown by Asha Bai *et al.* wherein they reported a 4.9% prevalence of known diabetes as well as an overall diabetes prevalence of 7.6% from different parts of Chennai.[36]

A study from Kashmir in adults above the age of 40 years reported a low prevalence of 4.25%.[35] The National Urban Diabetes Study (NUDS) from 6 major Indian cities showed an average prevalence of 12.6% among adults also reported a lower prevalence of diabetes compared to the studies from mega cities.[36] Our study shows that the number of diabetics in a similar age group (≥ 40 years) is much higher (623/3858) and the prevalence is about 16.2%.

## Conclusions

For this study, we used our community-based health surveillance data developed by us and maintained by the health centers. The number of individuals with diabetes living in the defined geographical area was identified and this was used to estimate the prevalence of diabetes. The limitation of this study is that no standard definition for diabetes based on laboratory reports could be ascertained as patients seek treatment from various health care providers, both in the public and private sectors. The diagnosis could not be confirmed by standard laboratory tests as these patients were undergoing treatment and it would have been expensive for each patient to undergo the diagnostic test for the purpose of the study.

We obtained data on the number of known diabetes (684) in the population of 11,835 between the ages of 0–80 years. The age-specific prevalence rates obtained were used to estimate the number of diabetic individuals living in Puducherry by using the age standardization method. It can be reasonably assumed that the total number of diagnosed and undiagnosed diabetics in the region would be at least twice this number, *i.e.,* about 100,000.[17,18]

The age and sex distribution shows that diabetes is more prevalent after the age of 40 years and therefore, this age group needs to be screened for undiagnosed diabetes. Screening the high-risk individuals first by applying the Indian Diabetes Risk Score (IDRS) followed by laboratory tests has been shown to be cost-effective in community-based screening programs for diabetes.[39]

The results of this screening should lead to further large scale epidemiological studies to build the evidence base. This will ascertain the magnitude of the rising epidemic which can then be halted and possibly reversed by concerted preventive measures adopted by public health policy makers through the active support of all the concerned stakeholders.
